# Repetitive H-Wave^® ^device stimulation and program induces significant increases in the range of motion of post operative rotator cuff reconstruction in a double-blinded randomized placebo controlled human study

**DOI:** 10.1186/1471-2474-10-132

**Published:** 2009-10-29

**Authors:** Kenneth Blum, Amanda LC Chen, Thomas JH Chen, Roger L Waite, B William Downs, Eric R Braverman, Mallory M Kerner, Stella M Savarimuthu, Nicholas DiNubile

**Affiliations:** 1 Department of Physiology & Pharmacology, Wake Forest University School of Medicine, Winston -Salem, North Carolina, USA; 2 Department of Engineering & Management of Advanced Technology, Chang Jung Christian University, Tainan, Taiwan, Republic of China; 3 Department of Occupational Health and Safety, Chang Jung Christian University, Tainan, Taiwan, Republic of China; 4 Department of Nutrigenomics, LifeGen, Inc. La Jolla, California, USA; 5 Orthopedic Surgeon, Havertown, Pennsylvania; and Department of Orthopaedic Surgery, Hospital of the University of Pennsylvania, Philadelphia, Pennsylvania, USA; 6 Department of Integrative Medicine, PATH Medical Clinics, and PATH Research Foundation, New York, New York, USA

## Abstract

**Background:**

Albeit other prospective randomized controlled clinical trials on H-Wave Device Stimulation (HWDS), this is the first randomized double-blind Placebo controlled prospective study that assessed the effects of HWDS on range of motion and strength testing in patients who underwent rotator cuff reconstruction.

**Methods:**

Twenty-two patients were randomly assigned into one of two groups: 1) H-Wave device stimulation (HWDS); 2) Sham-Placebo Device (PLACEBO). All groups received the same postoperative dressing and the same device treatment instructions. Group I was given HWDS which they were to utilize for one hour twice a day for 90 days postoperatively. Group II was given the same instructions with a Placebo device (PLACEBO). Range of motion was assessed by using one-way ANOVA with a Duncan Multiple Range Test for differences between the groups preoperatively, 45 days postoperatively, and 90 days postoperatively by using an active/passive scale for five basic ranges of motions: Forward Elevation, External Rotation (arm at side), External Rotation (arm at 90 degrees abduction), Internal Rotation (arm at side), and Internal Rotation (arm at 90 degrees abduction). The study also evaluated postoperative changes in strength by using the Medical Research Council (MRC) grade assessed strength testing.

**Results:**

Patients who received HWDS compared to PLACEBO demonstrated, on average, significantly improved range of motion. Results confirm a significant difference for external rotation at 45 and 90 days postoperatively; active range at 45 days postoperatively (p = 0.007), active at 90 days postoperatively (p = 0.007). Internal rotation also demonstrated significant improvement compared to PLACEBO at 45 and 90 days postoperatively; active range at 45 days postoperatively (p = 0.007), and active range at 90 days postoperatively (p = 0.006). There was no significant difference between the two groups for strength testing.

**Conclusion:**

HWDS compared to PLACEBO induces a significant increase in range of motion in positive management of rotator cuff reconstruction, supporting other previous research on HWDS and improvement in function. Interpretation of this preliminary investigation while suggestive of significant increases in Range of Motion of Post -Operative Rotator Cuff Reconstruction, warrants further confirmation in a larger double-blinded sham controlled randomized study.

## Background

No matter age, race, physical ability or activity levels, rotator cuff injuries are one of the most common causes of shoulder pain. In the United States alone, over 6 million people seek medical care each year for shoulder problems. Finding reliable treatment for pain and its fundamental causation, including pain reported by patients recovering from post-surgical rotator cuff reconstruction, presents a very real set of challenges.

### History and Treatment Aspects

For those less familiar with the field we are providing a brief statement relative to treatment options and outcomes for informative purposes only. Almost 100 years have past since the first report of rotator cuff repair in 1898 by W. Muller [1]. Today various solutions for difficult extended forms of rotator cuff lesions are available besides the closed and semi-closed arthroscopic techniques. It is inevitable in chronic degenerative tears with muscle atrophy that there will be loss of function. The treatment of massive rotator cuff tears must be adapted to the patient's individual needs and preoperative parameters to achieve the best outcome. Briefly, first the shoulder surgeon has to determine whether a direct transosseous repair is possible. If there is not enough remaining tissue, the tissue is atrophic, and the tendon stump can be reduced only with great tension, one can use a margin convergence technique for partial closure, perform a biceps tendoplasty, or perform local tendon transfers with subscapularis or infraspinatus muscle. Moreover, if the defect cannot be sufficiently closed, for example elderly patients with low demands can be treated with tubercleplasty/subacrominal decompression, whereas patients younger than 60 years with higher demands should receive muscle and tendon transfers. It is important that every effort should be made to perform early anatomic reconstruction in a young patients as well reduce pain as the function of the rotator cuff is of significant importance in the workforce. In younger patients for example, a balanced posterosuperior defect can be reconstructed by a deltoid muscle transfer, in contrast to an unbalanced one, which is best treated with an active transfer of the latissimus dorsi muscle and tendon. Generally, anterior defects can be addressed by pectorallis muscle transfer. However, if the humeral head is superiorly migrated, if signs of osteoarthritis are present, and if the patient is older than 70 years, a reverse prosthesis can be implanted as a salvage procedure. There have been many reviews pertaining to both treatment and clinical outcomes including management of isolated subscapularis tendon tears [2,3]; massive tears[4] and artroscopically assisted rotator cuff repair [5-7] as well as arthroscopic rotator cuff repair with double row fixation [8] Interestingly a prospective evaluation of the effect of rotator cuff integrity on the outcome of open cuff repairs found evidence to support open rotator cuff repair as an effective technique that restores excellent shoulder function[9].

### Anatomy

**Figure 1:**
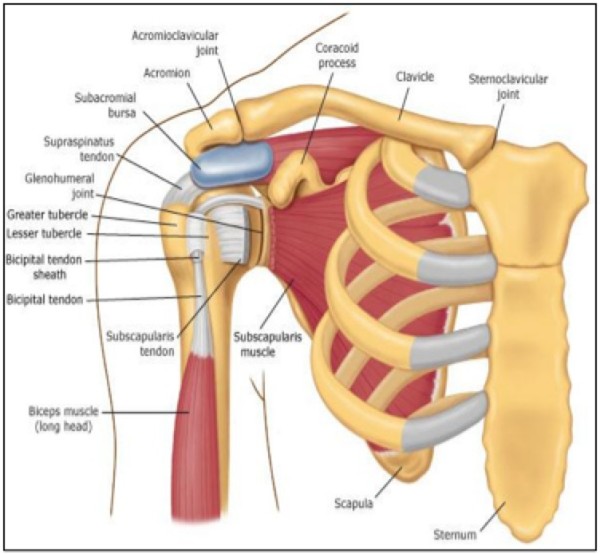
**Rotator cuff anatomy, anterior**.

**Figure 2:**
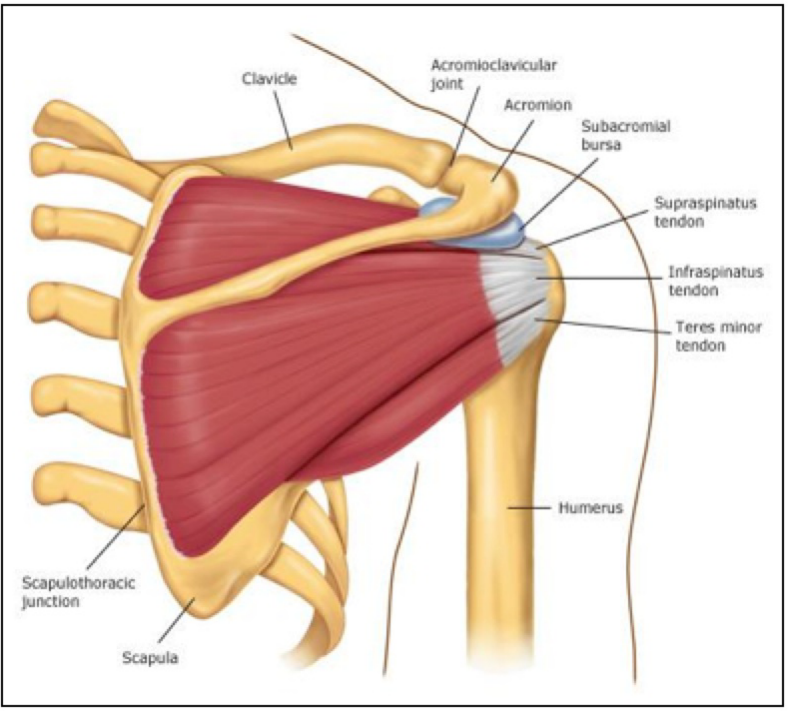
**Rotator cuff anatomy, posterior**.

The rotator cuff comprises of four small muscles and their musculotendinous attachments, acting as the dynamic stabilizer of the Glenohumeral joint. These muscles work as a complex, rather than individually. Often people injure one particular member of the rotator cuff, however most injuries usually involve more than one muscle (Figures 1 and 2). The subscapularis muscle is innervated by the subscapular nerve and originates on the scapula. It internally rotates the humerus and inserts on the lesser humeral tuberosity. The supraspinatus and infraspinatus are both innervated by the suprascapular nerve, originate in the scapula and insert on the greater tuberosity. Supraspinatus abducts the humeral head and acts as a humeral head depressor, while infraspinatus externally rotates and horizontally extends the humerus. The teres minor is innervated by the axillary nerve, originates on the scapula and inserts on the greater tuberosity, externally rotating and extending the humerus. The subacromial space lies underneath the acromion, the coracoid process, the acromioclavicular joint and the coracoacromial ligament. A bursa in the subacromial space provides lubrication for the rotator cuff. Figure 1 and figure 2 show the anterior and posterior rotator cuff anatomy, respectively.

The space between the undersurface of the acromion and the superior aspect of the humeral head is called the impingement interval. This space is normally narrow and is maximally narrow when the arm is abducted. Any condition that further narrows this space can cause impingement. Impingement can result from extrinsic compression or from loss of competency of the rotator cuff. Pain from any cause, such as overuse or injury, such as repetitive overhead motions from sporting activities, work tasks or daily chores, may lead to disuse or weakness of the cuff. The weakness results in cephalad migration of the humeral head due to loss of depressors.

### Critical Issues: Chronic illness and pain medications

The anatomy and biomechanics of the shoulder always guide the history and physical exam toward the appropriate diagnosis and treatment of rotator cuff injuries. There is still controversy over when Rotator Cuff surgery is recommended, According to **Bytomski & Black **[10] such, surgical management is usually reserved for refractory cases that have exhausted conservative measures, including regimens of nonsteroidal anti-inflammatory drugs (NSAIDs), corticosteroid injections, and functional rehabilitation therapy. In essence conservative measures are usually carefully assessed by the attending physician Several studies have documented high success rates following the surgical treatment of full-thickness rotator cuff tears of 1-5 cm. However, as Van Linthoudt *et al*. [11] found, a mean postsurgical symptom duration of 12 months (range, 3-48 months) and a mean time to recovery of shoulder power (75% of the value before the tear) as assessed by patients of 10 months [11] can be daunting, no matter the level of discomfort. Furthermore, only 25% of the patients studied by Van Linthoudt that underwent surgical treatment of full-thickness rotator cuff tears exhibited improved range of motion six years post surgery [11]. Historically, even less favorable and predictable results have been found in the treatment of massive tears (>5 cm) compared with small and medium sized tears; determining the most appropriate treatment for a patient with a massive rotator cuff tear can be challenging because of inconsistent outcome results. However, according to Jines and Savoie [12] more recent work using arthroscopic repair even of large and massive cuff tears results in good to excellent outcomes in 88% of patients. Moreover it is well known that not all massive rotator cuff tears have inconsistent outcome results. In fact, Kim *et al*. [13] concluded in their study that Arthroscopic repair of medium and large full - thickness rotator cuff tears had an equal outcome to technically unsuccessful arthroscopic repairs, which were salvaged by conversion to a mini -open technique. In fact, according to these researchers, surgical outcome depended on the size of the tear, rather than the method of repair.

Given the observed chronic nature of the recovery phase in patients undergoing surgical rotator cuff repair, it is important to note that chronic pain is consistently associated with disability and psychological distress [14]. In the WHO study, chronic pain sufferers were significantly more likely to experience marked limitations in activity and to have an anxiety or depressive disorder compared with patients without chronic pain [15]. In this regard, the management of pain and recovery of lost function associated with post operative rotator cuff reconstruction provides a significant challenge to the orthopedic surgeon.

Interestingly, preoperative patient expectation of the outcome for post operative rotator cuff reconstruction influences actual outcome. A rigorous multivariate analysis controlling for age, gender, smoking, Workers' Compensation status, symptom duration, number of previous operations, number of comorbidities, tear size, and repair technique confirmed that greater expectations were a significant independent predictor of both better performance at one year and greater improvement on all measures tested[16]. This observation, coupled with the known chronic nature of postoperative recovery from rotator cuff reconstruction, provided the impetus to develop a long term treatment and hands on patient compliance program through continual patient follow up and care. Thus simple therapy alone with any treatment modality, pharmacologically and or physical manipulation, must be coupled with moderate to strong patient interaction.

Treatment of patients with chronic pain, as often observed in rotator cuff injuries, usually involves prescription medication such as opioids, an approach that may reduce pain but that often fails to improve function, [17,18] and is also associated with significant adverse consequences such as opioid dependence, opioid-induced hyperalgesia, cognitive dysfunction, and suppression of the immune system [19-21]. Physical therapy and exercise programs may alleviate some types of pain, although compliance is often a problem [22-29]. There are 38,122 studies of pain related to postoperative procedures in PUBMED. Moreover, there are 30 studies specifically related to pain and rotator cuff reconstructive surgery.

#### H-Wave Device

During the past two decades, researchers have been increasingly interested in the control of pain and restoration of function through electrical stimulation. One area of this research has focused on the H-Wave^® ^(Electronic Waveform Lab, Inc, Huntington Beach, CA, USA) device. The purpose of the H-Wave device is to reduce or eliminate chronic pain and inflammation. This goal may be achieved via four mechanisms: firstly, through interstitial fluid shifts produced at very low frequencies (1-2 Hz) by direct stimulation of small-diameter skeletal muscle fibers and smooth muscles of the lymphatic system. This stimulation produces long rhythmical contractions of these specific muscle types, which can eliminate the accumulation of proteins that are a source of inflammation: an important component of pain and associated disability in patients with trauma or chronic injury [30]. Secondly, the H-Wave device also produces profound anaesthetic/analgesic effects when utilized at high frequencies (60 Hz) by affecting the function of the sodium pump within the nerve [31]. Thirdly, recent animal research has shown that stimulation of skeletal muscle by the H-Wave device produced a significant increase in the microcirculation, which was nitric oxide-dependent [32]. Fourth, repetitive HWDS to rodent hind limbs produced a profound and rapid increase in blood flow as a function of observed angiogenesis [33,34]. These two factors obviates the possibility that the repetitive HWDS reduces inflammation and promotes quicker healing and better recovery due to the elimination of protein build-up in post-operative conditions like rotator cuff reconstruction.

Recently our laboratory performed a meta-analysis to systematically review the efficacy and safety of the H-Wave device and program as a non-pharmacological analgesic treatment in chronic soft tissue inflammation and neuropathic pain. Five studies related to pain relief, reduction in pain medication and increased function obtained with the H-Wave device were included in the analysis. Data was analyzed using the random effects model, including adjustment to evaluate variability, size of study and bias in effect size. A total of 6535 participants were included in the meta-analysis [35-40]. The findings indicate a moderate to strong effect of the H-Wave device in providing pain relief, reducing the requirement for pain medication and increasing function. The most robust effect was observed for improved function, suggesting that the H-Wave device may facilitate a quicker return to work and other related daily activities [40].

### Rationale for Pain Reduction

Pain may be undertreated, contributing to anguish, as reported by the World Health Organization. Pain may also be over treated, inadvertently contributing to drug addiction, drug diversion, and even death. Thus, primum non nocere--*first, do no harm*--is not easily achieved in the pharmacological treatment of pain, particularly in pain reported chronically. In 2008, Henn *et al*. [41] concluded in a perspective study of 125 patients with Workers' Compensation claims report worse outcomes, even after controlling for confounding factors (1.e. age, work demands, lower marital rates, education levels, preoperative expectations) compared to non-workers' compensation patients. Thus since the study by Henn *et al*. provides evidence that the existence of a Workers' Compensation claim portends a less robust outcome following rotator cuff repair stimulated interest in evaluating H-Wave Device Stimulation (HWDS)to improve outcome results. Moreover even with today's ultra technical sophistication according to Kasten *et al*. [42] surgery of the shoulder can cause considerable pain. According to data from randomized controlled trials, local or regional anaesthesia is recommended for analgesia during and after surgery of the upper extremity. This treatment involves potent addictive opioids and non-steroidal anti-inflammatory drugs in a multimodal analgesia approach. Additionally since the pain is profound according to a meta-analysis of randomized controlled trials, an interscalene block is recommended for analgesia during and after surgery of the shoulder. Other recommendations include physiotherapy postoperatively. Interestingly, while the use of arthroscopic procedures for most knee conditions yields relatively mild and controlled pain, it is known that arthroscopic procedures for rotator cuff repair and reconstruction induces more significant pain for the patient during the recovery phase, and hence remains a great challenge. The advent of pain pumps was initially met with enthusiasm by many shoulder surgeons, but has led to serious complications involving chodrolysis. In fact, several studies which were confirmed by a bovine and rabbit cartilage study suggested that there is significant chondrotoxicity from bupivacane, a local anesthetic commonly used in pain pumps [43].

With this in mind our laboratory, over last two decades, has been searching for a non-pharmacologic alternative to manage pain and restore lost function associated with acute, subacute and chronic stages of various injuries and conditions, as well as pain and lost function associated with postoperative recovery and rehabilitation. The limited options for the management of soft tissue inflammation, neuropathic pain and in particular pain derived from rotator cuff injuries have prompted the search for more effective therapies; the following study is one example of that arduous search.

## Methods

### Patient selection

All of the 22 patients had moderate to severe rotator cuff injuries of various origin. All patients signed standard approved IRB consent forms. This study received IRB approval from the orthopedic clinic and a follow up approval for the development of the actual write up the PATH Research Foundation, New York, New York. The registration number of the IRB NIH registration is # (IRB00002334). In this study there were 9 males and 13 females.

### Patient inclusion criteria

In the present investigation the following inclusion criteria was developed which provided consistent eligibility criteria for enrollment into the study:

1. The patients had a pre-diagnosis of rotator cuff injury sufficient to require shoulder rotator cuff reconstruction surgery.

2. The patient could be either male or female between the ages of 18-75 years of age.

3. The patient is in satisfactory health as determined by the investigator on the basis of medical history and physical examination.

4. The patient provided a written informed consent approved by the IRB prior to admission to the study.

### Protocol

Albeit other prospective randomized controlled clinical trials on repetitive HWDS, this is the first randomized double-blind HWDS/Placebo controlled prospective study executed at a well known orthopedic clinic and hospital in Inglewood, California. The study assessed the effects of HWDS on range of motion, and strength testing in 22 patients who underwent rotator cuff reconstruction. Each patient admitted into the study was assessed for both range of motion by the pre-angles of ROM effects and strength of testing. The purpose of this study was to determine differences between groups of patients who underwent rotator cuff reconstruction: 1) H-Wave Electro stimulation (Electronic Waveform Lab. Huntington Beach, CA) [HWDS], and 2) Sham-Placebo device (PLACEBO). Each group was instructed to use their device for one hour twice a day, for 90 days; starting the day of surgery. For this experiment the sham group was told not to expect any sensation from the device (like a micro-current device). All patients were randomly assigned by RW into one of the two groups. The only individual knowledgeable about which patient received either HWDS or PLACEBO was the proctor RW. No other staff member of the team knew the code or the results to avoid any bias. At the end of the study the data was submitted to a staff member for statistical evaluation. The blind procedure was preserved throughout the entire study. This was validated by RW who interviewed each staff member to validate blinding. Each patient was carefully fitted and instructed by RW with HWDS and or PLACEBO. The patients were instructed to use the device twice daily for one hour throughout the 90 day experimental period. It is noteworthy that electrode placement for the H-Wave device was inserted by the orthopedic surgeon around the surgery site during the operative procedure. Thus, each patient had their sterile pad placement carefully fitted by the attending orthopedic surgeon at the surgery site directly after the operative sutures were in place and before the dressings were applied. An H-Wave two channel (A & B) was used as the testing device. Channel A electrode placement: Superior pad was placed at the superior angle of the scapula; overlapping the middle fibers of the Trapezius and origin of the Supraspinatus muscle. Inferior pad was placed just superior to the Deltoid Tuberosity on the Humerus bone. Channel B electrode placement: Anterior and posterior pads were placed at the beginning and end of the suture line of the open reduction surgery. This reduced stress relative to having to place the electro pads every day for the first week. Every patient was visited again on a two week basis and instructions were reviewed if needed by RW with HWDS and or PLACEBO. If any problems arose while the patient was on the surgical floor of the Hospital they were given a 24 hour phone number to call if needed. It is noteworthy that there were no surgical complications for any enrolled study member. Range of motion was assessed for differences between the groups preoperatively, 45 days postoperatively, and 90 days postoperatively by using an active/passive scale for a number of basic ranges of motions: *Forward Elevation*, *External Rotation *(arm at side), *External Rotation *(arm at 90 degrees abduction), *Internal Rotation *(arm at side), and Internal Rotation (arm at 90 degrees abduction). The study by also using the Medical Research Council (MRC) grade assessed strength testing. All of the patients in the study underwent open Rotator Cuff repair and/or reconstruction. I n fact all of the patients meeting inclusion criteria received major open rotator cuff repair and or reconstruction rather than arthroscopic procedure. None of the patients in the study had pain pumps. It was important that there was concealment of group allocation to prevent group interaction and potential bias. It is noteworthy that these patients were all selected and enrolled by assessing suitability for the study within a very short time frame (within days). However, there was no pre-selection in terms of group entry. Thus entry of a subject to a group was purely randomized. The main inclusion criteria on the acceptance of surgical patients was that the tear had to be large enough to justify an open reduction surgery and not an Arthroscopic repair. For this study the measurement of the tear and severity were not considered as inclusion data per se.

### Post-operative follow-up

All of the patients had the same post-operative follow-up protocol and the operation and follow-up procedures were all performed by the same surgeon to provide continuity of the study. In terms of post-operative rehabilitation it is standard procedure that in post-operative rotator cuff reconstruction patients are not allowed to lift the arm and they are fitted for a sling for a long period of time. In this study, no active Physical Therapy (PT) occurred in most cases for at least 8 weeks post surgery. It was the policy of the attending physician that no active Physical therapy was to be performed inside of 8 weeks post operative period. After the 8 weeks Physical Therapy was encouraged and began. However each patient utilized the H-Wave device and program or sham and were allowed only passive Range of Motion (ROM). Moreover only the indirect signs of recovery were measured. For this study RW used Joint Range of Motion and Muscle strength. While there was pre-diagnosis of the tear by MRI in general there was no post MRI's performed. This was the decision of the attending surgeon due to inconsistency of post MRIs. Furthermore, the patient was asked to perform HWSD treatments one hour twice daily for one month (active post operative treatment-barely visible muscle contraction), thereafter they were to reduce treatment to one treatment for one hour every day for 2 months (active therapy-high muscle contraction or to tolerance). It is noteworthy that each patient had reporting sheets (Diary) to record their treatments. This provided a means to determine compliance.

## Statistics

Differences between age at surgery, affected side, and gender, were determined using a Chi-square test. Statistical analysis of the range of motion preoperatively, 45 days postoperatively, and 90 days postoperatively was determined by using an Active/Passive scale. The range of motion of the involved extremity was compared to that of the uninvolved extremity by using a One-Way ANOVA with a Duncan Multiple Range Test. The MRC range scale assessed strength testing between the groups. Statistical analysis employed the statistical package SAS [44]. Careful consideration as to the objectiveness of the measure for ROM was taken into account prior to development of the protocol for this study. The measurement used has been validated in other studies, was familiar to the attending physician and was objective.

## Results

**Figure 3:**
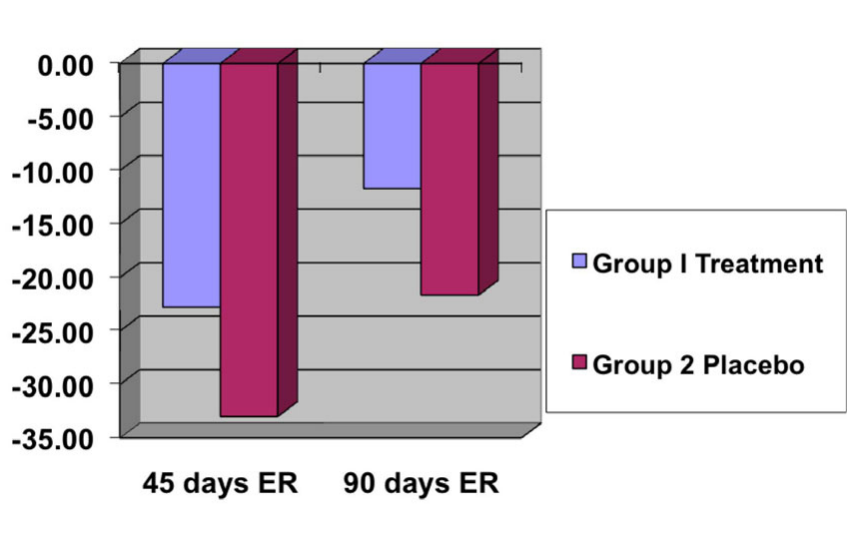
**45 and 90 day data showing the comparative changes (improvements) in Post Operative loss of motion (in degrees) for External Rotation (ER) of the afflicted arm/shoulder between Group 1 (45 days: p = 0.007; 90 days: p = 0.007) and Group 2 (45 days: p = 0.007; 90 days: p = 0.007)**.

**Figure 4:**
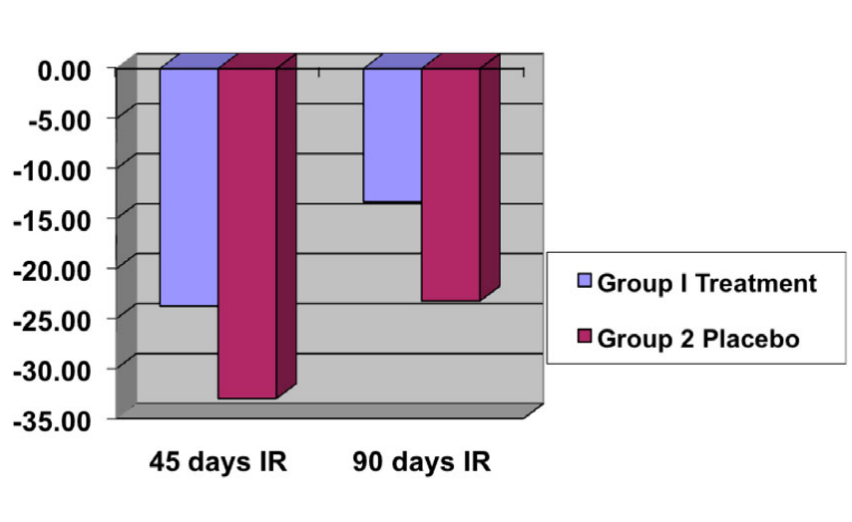
**45 and 90 day data showing the comparative changes (improvements) in Post Operative loss of motion (in degrees) for Internal Rotation (IR) of the afflicted arm/shoulder between Group 1 (45 days: p = 0.007; 90 days: p = 0.006) and Group 2 (45 days: p = 0.007; 90 days: p = 0.006)**.

A total of 22 patients were studied following rotator cuff surgery. 12 patients received an H-Wave Electro Stimulation device (Group 1), and 10 patients received a Placebo device (Group 2). There were no differences between the groups in age, gender, or side of surgery. There was no significant difference between the two groups for strength testing; but generally, the 12 patients in Group 1 who received an active device showed a non-significant higher strength level postoperatively. Group I patients showed on average a significant amount of increased range of motion. A significant difference was found for external rotation at 45 and 90 days postoperatively. At 45 days postoperative (PO) Group 1 had a loss of 22.75 degrees (p = 0.0079) in external rotation (arm at side), while Group 2 had a loss of 33.00 degrees (p = 0.007) (see figure 3). At 90 days postoperative (PO) Group 1 had a loss of 11.67 degrees (p = 0.007) in external rotation (arm at side), while Group 2 had a loss of 21.65 (p = 0.007). Group 1 also showed significant difference with internal rotation (arm at 90 degrees) at 45 days postoperatively with a loss of 23.75 degrees (p = 0.007), while Group 2 had a loss of 33.00 degrees (p = 0.007). 90 days postoperatively Group I had a loss of 13.33 degrees (p = 0.006), while Group 2 had a loss of 23.25 degrees (p = 0.0062) (see figure 4). All other range of motions showed no statistical difference.

It is noteworthy, after the initiation of Physical Therapy in both active and non- active HWDS (post 8 weeks of surgery) the patients that had active H-Wave advanced more quickly on Physical Therapy as measured by ROM and flexibility compared to the non -HWDS. Interestingly, following the approval of the attending physician, for the H-Wave group a number of patients started Physical Therapy at the 6 week time frame but this quicker Physical therapy did not occur with the sham subjects.

## Discussion

Albeit the small sample size, statistical analysis has shown that patients who have undergone rotator cuff reconstruction and used HWDS daily compared to PLACEBO (SHAM) postoperatively, have benefited by increased range of motion and possibly strength.

In physical medicine trials such as with the H-Wave device, randomized, blinded, placebo controlled studies are very difficult to produce and control in comparison with other modalities and/or pharmaceutical trials [45]. Being cognizant of this fact, one of us (RW) carefully developed a protocol that allowed for hands on blinded controlled procedures. In each case and for every individual rigorous over site was systematically maintained throughout the HWDS and PLACEBO periods. However, despite these difficulties, the randomized double-blinded Placebo controlled study herein presented on the H-Wave device provides additional evidence that studies of this nature can be successfully executed.

Additional studies, involving a much larger population, are currently in progress and should provide additional useful information. In future studies we expect that not only range of motion would be significantly impacted but so will strength, whereby we have already seen in the present study a positive, but not a statistically-significant trend.

The current significant results of daily repetitive long term HWDS and program suggest an improved range of motion and possibly strength post operatively. The mechanism of such a benefit may reside in the importance of nitric oxide in cell communication and the inflammatory process [46]. Wound healing impairment represents a particularly challenging clinical problem to which no efficacious treatment regimens currently exist. The factors ensuing appropriate intercellular communication during wound repair are not completely understood. Although protein-type mediators are well- established players in the process, emerging evidence from both animal and human studies indicate that nitric oxide plays a key role in wound repair. For example, nitric oxide elicits functional MMP-13 protein-tyrosine nitration during wound repair [47]. The beneficial effects of nitric oxide on wound repair may be attributed to its functional influences on angiogenesis [48,34], inflammation, cell proliferation, matrix deposition, and remodeling [49].

The fact that animal research has shown that repetitive HWDS induces significant angiogenesis compared to single or intermittent HWDS suggest that this finding may induce healing in tissue tears. Muscular contraction or shear-wall stress is the best known factor for the intrinsic production of angiogenesis. Interestingly, by stimulating slow twitch myofibers, with associated mitochondria activity, a larger and denser network of angiogenesis will be formed. It is noteworthy, unlike other electrotherapeutic devices which utilize fast twitch muscle fibers (e.g. TENS, EMS, Hi-Volt Galvanic, interferential), H-Wave therapy is known to utilize these slow twitch muscle fibers in a non-fatiguing contraction [31].

A limitation in the present study is that the number of patients evaluated, although significantly different, is small and thus cautious interpretation may be suggested until the data can be confirmed in a larger similar study. Moreover, appropriate stratification was difficult to achieve because of the small number of patients. While size of the tear was assessed as well as pre-operative measurement of range of motion the groups were randomized rather than preselected. On one hand this allows for a better double-blinded study but on the other hand it does not control for appropriate stratification. Another concern due to a small population is that rotator cuff injuries are not clearly defined and classified with respect to localization, size and chronic/acute tears. Indeed we are cognizant that large subscapularis tears for example will have a different outcome in postoperative range of motion than supaspinatus tears. It is noteworthy that although the actual percentage of individuals that complied with the treatment request was not adequately measured in the sham group it is of importance that 100% of individuals in the HWDS group utilized their device until the end of the study (compliance in this group was accessed to be 85%). This is compared to a 40% return of the H-Wave device from the sham group during the last 30 days of the study days (P = 0.029) using a Fisher exact test. We are cognizant that while there are no published studies to our knowledge that have reported 100% of all rotator cuffs had healed our results only support indirect measurements to access function not overall repair. While the funding for this study was d erived from a source that would benefit financially from results, it is imperative to realize that no one in the executive level provided significant input to the outcome results of the study. With that said one could still argue potential bias albeit the real likelihood of such bias.

Shoulder surgeries notoriously have tremendous issues with pain in the first few weeks. Almost none of the active H-Wave patients used pain medication while in the hospital during their treatment. While the study didn't directly assess pain, the improvement of ROM certainly suggests that the H-Wave device and program had significant pain relieving effects in these patients. However we cannot draw a definitive conclusion concerning pain relief from the present study. Additional work in this regard is warranted.

Moreover, frozen shoulders are a major problem, in post operative follow-up. It has been regarded by some as an enigma, so catching the range of motion early is very valuable [50]. This is the reason why we set up H-Wave and Sham in the recovery room. Based on our earlier research on microcirculation in rats [34], we are confident that utilization of the H-Wave device early on induced fluid shifts, increased blood flow dependent on NO and potentially initiated the angiogenesis process. Moreover it is well known that inactivity of the shoulder in some will result in soft tissue fibrosis and joint contracture following anterior acromioplasty and/or rotator cuff repair. The rehabilitation process is usually delayed for up to four to six weeks to allow for healing [51]. There have been recommendations that following surgery of the shoulder that active -assisted shoulder motion should be encouraged immediately. In our study, while Physical Therapy was not allowed for most patients to begin until 8 weeks post surgery, it was noted that the patients receiving HWDS compared HWDSHAM, began to move in a micro-way earlier under the sling. It is hereby conjectured not proven that this phenomena may have been due to the physiological proven effects of the H-Wave device. Thus, in terms of augmenting the healing process by virtue of increased NO-dependent microcirculation and angiogenesis following chronic HWDS treatment these benefits improved outcome [31-34]. Certainly, this warrants more extensive investigation.

These findings in this preliminary investigation suggest but do not mandate that HWDS compared to PLACEBO induces a significant and robust increase in range of motion in postoperative management of rotator cuff Reconstruction. Thus, using the H-Wave device if confirmed in a larger study, can be beneficial for the management of post operative rotator cuff Reconstruction in terms of significantly increasing their range of motion, function and possibly strength, which will ultimately lead to a faster healthier recovery.

## Conclusion

Given any bias and or limitations described herein, confirmation of these results in a larger randomized double-blind sham controlled study will provide impetus to utilize HWDS as a frontline analgesic alternative increasing function and achieving better recovery outcomes.

## Competing interests

KB and NDN are paid consultants of Electronic Waveform Lab, Huntington, Beach, California. RW was a paid consultant during the performance of this study.

## Authors' contributions

KB was the major investigator and drafted the manuscript. ALC was a co-author and contributed to the writing of the manuscript. TJHC was a co-corresponding author and contributed to the writing of the manuscript. RW conducted the clinical aspects of the investigation. BWD was a co-author who contributed to the writing of the manuscript. ERB was involved in the IRB approval and contributed to the editing of the manuscript. MMK contributed to the writing, editing, and literature referencing for the manuscript. SMS was involved in the references and literature search. ND was a co-author who contributed to the overall draft of the manuscript.

All authors read and approved the final version of the manuscript.

## Pre-publication history

The pre-publication history for this paper can be accessed here:



## References

[B1] Habermeyer P (1995). Open surgical therapy of the rotator cuff. Orthopade.

[B2] Barti C, Imhoff AN (2007). Management of isolated subscapularis tendon tears. Orthopade.

[B3] Edwards TB, Walch G, Sirvaux F, Mole D, Nove-Josserand L, Boulahla A, Neyton L, Szabo I, Lindgre B, O'Connor DP (2006). Repair of teras of the subscapularis surgical technique. J Bone Joint Surg Am.

[B4] Vandenbussche E, Bensaida M, Mutschler C, Dart T, Augereau B (2004). Massive tears of the rotator cuff treated with a deltoid flap. Int Orthop.

[B5] Bozzotta H, Prunner K (2004). Arthroscopically assisted rotator cuff repair. Arthroscopy.

[B6] Petit C, Millett PJ (2008). Arthroscopic removal of endoButton after anterior cruclate ligament reconstruction : case report and surgical technique. Am J Orthop.

[B7] Millett PJ, Braun S (2009). The "bony Bankart bridge" procedure : a new arthroscopic technique for reduction and internal fixation of a bony Bankart lesion. Arthroscopy.

[B8] Huijsmans PE, Pritchard MP, Berghs BM, van Rooyen KS, Walalce AL, d Beer JF (2007). Arthropscopic rotator cuff repair with double -row fixation. J Bone Joint Surg Am.

[B9] Klepps S, Bishop J, Lin J, Cahlon O, Strauss A, Hayes P, Flatow EL (2004). Prospective evaluation of the effect of rotator cuff integrity on the outcome of open rotator cuff repairs. Am J Sports Med.

[B10] Bytomski JR, Black D (2006). Conservative treatment of rotator cuff injuries. J Surg Orthop Adv.

[B11] Van Linthoudt D, Deforge J, Malterre L, Huber H (2003). Rotator cuff repair. Long-term results. Joint Bone Spine.

[B12] Jones C, Savoie FH (2003). Arthroscopic repair of large and massive rotator cuff tears. Arthroscopy.

[B13] Kim SH, Ha KI, Park JH, Kang JS, OH SK, OH I (2003). Arthroscopic versus mini-open salvage repair of the rotator cuff tear: outcome analysis at 2 to 6 years' follow -up. Arthroscopy.

[B14] Khouzam RH (2000). Chronic pain and its management in primary care. South Med J.

[B15] Gureje O, Von Korff M, Simon GE, Gater R (1998). Persistent pain and well-being: a World Health Organization study in primary care. JAMA.

[B16] Tunks ER, Crook J, Weir R (2008). Epidemiology of chronic pain with psychological comorbidity: prevalence, risk, course, and prognosis. Can J Psychiatry.

[B17] Eriksen J, Sjøgren P, Bruera E, Ekholm O, Rasmussen NK (2006). Critical issues on opioids in chronic non-cancer pain: an epidemiological study. Pain.

[B18] Højsted J, Sjøgren P (2007). An update on the role of opioids in the management of chronic pain of nonmalignant origin. Curr Opin Anaesthesiol.

[B19] Eisenberg E, McNicol ED, Carr DB (2005). Efficacy and safety of opioid agonists in the treatment of neuropathic pain of non-malignant origin: systematic review and meta-analysis of randomized controlled trials. JAMA.

[B20] Furlan AD, Sandoval JA, Mailis-Gagnon A, Tunks E (2006). Opioids for chronic noncancer pain: a meta-analysis of effectiveness and side effects. CMAJ.

[B21] Noble M, Tregear SJ, Treadwell JR, Schoelles K (2008). Long-term opioid therapy for chronic noncancer pain: a systematic review and meta-analysis of efficacy and safety. J Pain Symptom Manage.

[B22] Fass A, Van Eiijk JTM, Chavannes AW, Gubbels JW (1995). A randomized trial of exercise therapy in patients with acute low back pain: efficacy on sickness absence. Spine.

[B23] Schonstein E, Kenny D, Keating J, Koes B, Herbert RD (2003). Physical conditioning programs for workers with back and neck pain: a Cochrane systematic review. Spine.

[B24] Wetzel FT, McNally TA, Phillips FM (2002). Intradiscal electrothermal therapy used to manage chronic discogenic low back pain. Spine.

[B25] North RB, Wetzel FT (2002). Spinal cord stimulation for chronic pain of spinal origin: a valuable long-term solution. Spine.

[B26] Maurer P, Block JE, Squillante D (2008). Intradiscal electrothermal therapy (IDET) provides effective symptom relief in patients with discogenic low back pain. J Spinal Disord Tech.

[B27] Andersson GB, Mekhail NA, Block JE (2006). Treatment of intractable discogenic low back pain. A systematic review of spinal fusion and intradiscal electrothermal therapy (IDET). Pain Physician.

[B28] Freeman BJ, Fraser RD, Cain CM, Hall DJ, Chapple DC (2005). A randomized, double-blind, controlled trial: intradiscal electrothermal therapy versus Placebo for the treatment of chronic discogenic low back pain. Spine.

[B29] Kumar K, Taylor RS, Jacques L, Eldabe S, Meglio M, Molet J, Thomson S, O'Callaghan J, Eisenberg E, Milbouw G (2007). Spinal cord stimulation versus conventional medical management for neuropathic pain:a multicentre randomised trial in patients with failed back surgery syndrome. Pain.

[B30] Blum K, Chen JHT, Ross BD (2005). Innate properties of H-Wave device, a small fiber stimulator provides the basis for a paradigm shift of electro-therapeutic treatment of pain with increased functional restoration associated with human neuropathies. Townsend Letter for Doctors and Patients.

[B31] Blum K, Chen TJ, Ross BD (2005). Innate properties of H-Wave device, a small fiber stimulator provides the basis for a paradigm shift of electro-therapeutic treatment of pain with increased functional restoration associated with human neuropathies by affecting tissue circulation: a hypothesis. Med Hyp.

[B32] Blum K, Ho CK, Chen LC, Chen, Fulton M, Fulton B, Westcott W, Reinl G, Braverman ER, Dinubile N, Chen TJH (2008). The H-Wave device induces NO-dependent augmented microcirculation and angiogenesis, providing both analgesia and tissue healing in sports injuries. The Physician and Sportsmedicine.

[B33] Smith TL, Blum K, Callahan MF, DiNubile NA, Chen TJ, Waite RL (2009). H-Wave induces arteriolar vasodilation in rat striated muscle via nitric Oxide-mediated mechanaisms. J Orthop Res.

[B34] Smith TL, Blum K, Waite RL, Heaney WJ, Callahan M (2007). The microvascular and hemodynamic mechanisms for the therapeutic actions of H-Wave muscle stimulation. 6th Combined Meeting of the Orthopaedic Research Societies, 21 October Honolulu, Hawaii.

[B35] Kumar D, Marshall HJ (1997). Diabetic peripheral neuropathy: amelioration of pain with transcutaneous electrostimulation. Diabetes Care.

[B36] Kumar D, Alvaro MS, Julka IS, Marshall HJ (1998). Diabetic peripheral neuropathy: effectiveness of electrotherapy and amitriptyline for symptomatic relief. Diabetes Care.

[B37] Julka IS, Alvaro M, Kumar D (1998). Beneficial effects of electrical stimulation on neuropathic symptoms in diabetes patients. J Foot Ankle Surg.

[B38] Blum K, Dinubile N, Chen TJH, Waite RL, Schoolfield J, Martinez-Pons M, Callahan MF, Smith TL, Mengucci J, Blum SH, Meshkin B (2006). H-Wave, a nonpharmacologic alternative for the treatment of patients with chronic soft tissue inflammation and neuropathic pain: a preliminary statistical outcome study. Adv Ther.

[B39] Blum K, Martinez-Pons M, Dinubile NA (2006). H-Wave device, a small musclefiber stimulator, significantly reduces pain medication, increases function and improves health in 6,774 patients with chronic soft tissue injuries and/or neuropathic pain: an extended population observational study. Adv Ther.

[B40] Blum K, Chen AL, Chen TJ, Prihoda TJ, Schoolfield J, Dinubile N, Waite RL, Arcuri V, Kerner M, Braverman ER, Rhoades P, Tung H (2008). The H-Wave(R) device is an effective and safe non-pharmacological analgesic for chronic pain: a meta-analysis. Adv Ther.

[B41] Henn RF, King L, Tashijan RZ, Green A (2008). Patients with workers' compensation claims have worse outcomes after rotator cuff. J Bone Joint Surg AM.

[B42] Kasten P, Christian JP, Volk T, Schmelzer-Schmied N (2008). Analgesia in shoulder, elbow and hand surgery. Orthopede.

[B43] Busfield BT, Romero DH (2009). Pain pump use after shoulder arthroscopy as a cause of glenohumeral chondrolysis. Arthroscopy.

[B44] Cohen J (1988). Statistical Power Analysis for the Behavioral Sciences.

[B45] Zambito A, Bianchini D, Gatti D, Viapiana O, Rossini M, Adami S (2006). Interferential and horizontal therapies in chronic low back pain: a randomized double blind, clinical study. Clin Exp Rheumatol.

[B46] Ferreira AA, Kwasniewski FH, Delani TC, Torres MG, Silva MA, Caparroz-Assef SM, Cuman RK, Bersani-Amado CA (2007). Acute immune and non-immune inflammatory response in spontaneously hypertensive rats and normotensive rats. Role of endogenous nitric oxide. Inflammation.

[B47] Lizarbe TR, García-Rama C, Tarín C, Saura M, Calvo E, López JA, López-Otín C, Folgueras AR, Lamas S, Zaragoza C (2008). Nitric oxide elicits functional MMP-13 protein-tyrosine nitration during wound repair. FASEB J.

[B48] Fukumura D, Gohongi T, Kadambi A, Izumi Y, Ang J, Yun CO, Buerk DG, Huang PL, Jain RK (2001). Predominant role of endothelial nitric oxide synthase in vascular endothelial growth factor-induced angiogenesis and vascular permeability. Proc Natl Acad Sci USA.

[B49] Sanders-Williams R, Annex BH (2004). Plasticity of Myocytes and Capillaries. Circ Res.

[B50] Dodenhoff RM, Levy O, Wilson A, Copeland SA (2000). Manipulation under anesthesia for primary frozen shoulder: effect on early recovery and return to activity. J Shoulder Elbow Surg.

[B51] Guven Z (2003). Rehabilitation following anterior acromioplasty. Acta Orthop Traumatol Turc.

